# Supplementary material for: Age Estimate of GJB2-p.(Arg143Trp) Founder Variant in Hearing Impairment in Ghana, Suggests Multiple Independent Origins across Populations
Source: Biology (Basel). 2022 Mar 21;11(3):476. doi: 10.3390/biology11030476 (PMC8945073; doi:10.3390/biology11030476)
Supplement: Supplementary file 1 [file biology-11-00476-s001.zip › biology-1631172-supplementary.pdf]

Supplementary Materials

Table S1. Quality metrics of markers selected for age estimation and haplotype analysis

Marker	Chr: Pos	MQ	DP	AvGQ	VQSLOD	FILTER	BIOTYPE
**rs115802719**	13:20635310	249.92	9622	89.78	7.35	PASS	Exonic
**rs7324021**	13:20637140	250	7530	78.15	4.98	PASS	Intronic
**rs17075877**	13:20641422	248.84	9149	85.73	5.72	PASS	Exonic
***rs80338948**	13:20763294	249.37	13226	88.56	5.1	PASS	Exonic
**rs9578260**	13:20763754	249.44	17623	79.17	6.77	PASS	Intronic

Chr:Pos=Chromosome and base pair position; MQ=Mapping quality; DP=Depth of coverage; AvGQ=Average genotype quality; VQSLOD=Variant quality score log of the odds; * = Disease allele

Table S2. Quality metrics of imputed SNVs markers and accuracy scale

Marker	Chr:Pos	Ref/Alt	Gene	Bio-type	LD (r^2)	Genetic Map (cM)	MAF (1KGP3 AFR populations)	Imputation accuracy (scale: 0 - 1)
**rs7329857**	13:20762929	G/A	*GJB2*	3'UTR	0.38	0.000000	A= 0.2196	0.85
**rs7337074**	13:20762936	T/A	*GJB2*	3'ÚTR	0.38	0.000007	A = 0.0002	0.85
***rs80338948**	13:20763294	G/A	*GJB2*	Exonic	1.000	0.000365	A= 0.0002	1
**rs9578260**	13:20763754	G/A	*GJB2*	In-tronic	0.38	0.000825	A = 0.0002	1
**rs9578261**	13:20764147	C/A	*GJB2*	In-tronic	0.36	0.001218	T = 0.0002	0.99
**rs9579800**	13:20764312	T/A	*GJB2*	In-tronic	0.36	0.001383	A = 0.0002	0.99
**rs74035963**	13:20764722	A/G	*GJB2*	In-tronic	0.36	0.001793	G = 0.0002	0.99
**rs74035964**	13:20764815	T/C	*GJB2*	In-tronic	0.36	0.001886	C = 0.0002	0.99
**rs74035965**	13:20764850	C/T	*GJB2*	In-tronic	0.36	0.001921	T = 0.0002	0.99

Computed genetic map distance in centimorgans (cM), and minor allele frequencies (MAF) in the reference 1000 Genome reference panel, phase 3, version 5 database (1KGP3v5); Computed linkage disequilibrium (r2) for each marker respectively. RSID=reference SNP ID; Chr:Pos=Chromosome and base pair position; Ref/Alt=Reference and alternate alleles; LD=linkage disequilibrium; cM=Centimorgan; AAF=alternate allele frequency; * = the disease mutation

Table S3. Haplotypes and haplotype frequencies in Ghanaian p.R143W-positive and p.R143W-negative populations based on five markers that were in linkage disequilibrium with p.R143W.

Marker and Ref/Alt allele					Hap	Hap Name	Frequency		
rs115802719	rs7324021	rs1705877	rs80338948	rs9578260	-	-	GHA-38	R143W-1	R143W-2
A/G	T/G	A/G	G/A	G/A	-	-			
0	0	0	0	0	00000	Hap1	0.57	0	0
0	0	0	0	1	00001	Hap2	0.26	0	0
0	0	0	1	0	00010	Hap3	0	0.067	0.067
0	0	0	1	1	00011	Hap4	0	0.333	0.33
0	0	1	0	1	00101	Hap5	0.026		0
0	0	1	1	1	00111	Hap6	0	0.067	0.067
0	1	0	0	0	01000	Hap7	0.079	0	0
0	1	0	0	1	01001	Hap8	0.026	0	0
0	1	0	1	0	01010	Hap9	0	0.033	0
0	1	0	1	1	01011	Hap10	0	0.3	0.33
1	0	1	0	0	10100	Hap11	0.026	0	0
1	0	1	0	1	10101	Hap12	0.013	0	0
1	0	1	1	1	10111	Hap13	0	0.2	0.2

The "0s" and "1s" against each marker indicate whether the marker is homozygous for the reference allele (0) or homozygous for the alternate allele (1). Ref/Alt=Reference and alternate alleles; Hap = Haplotype.

Table S4. Excel 1KGP3v5 WES haplotype frequencies and diversity (excel spreadsheet attached).

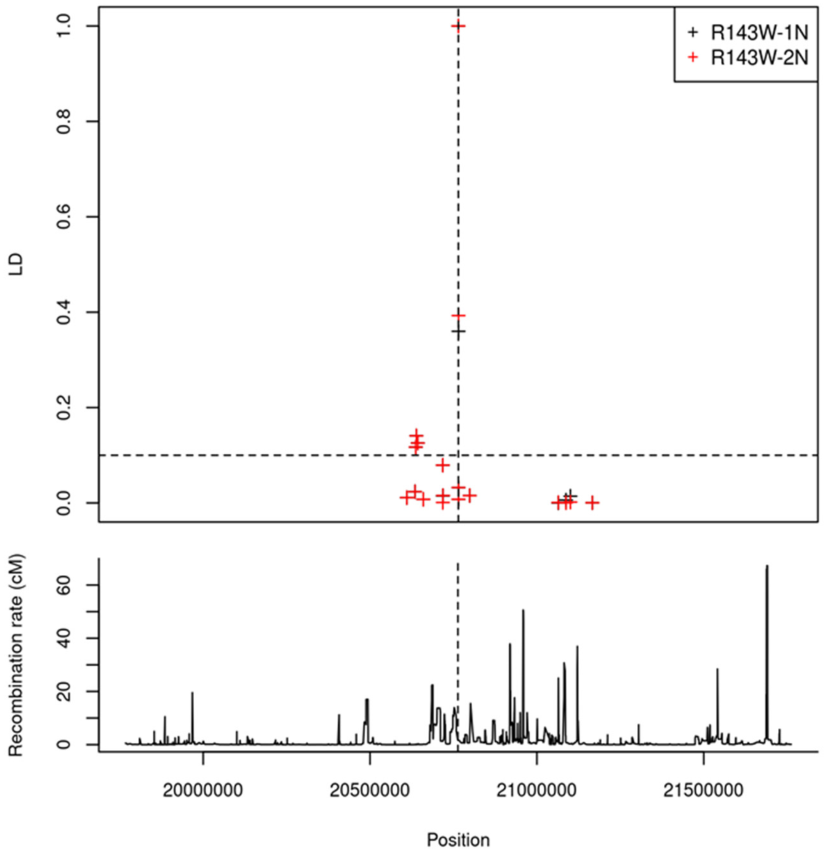

Figure S1: Pattern of linkage disequilibrium and recombination in the 2 Mb region around p.R143W.

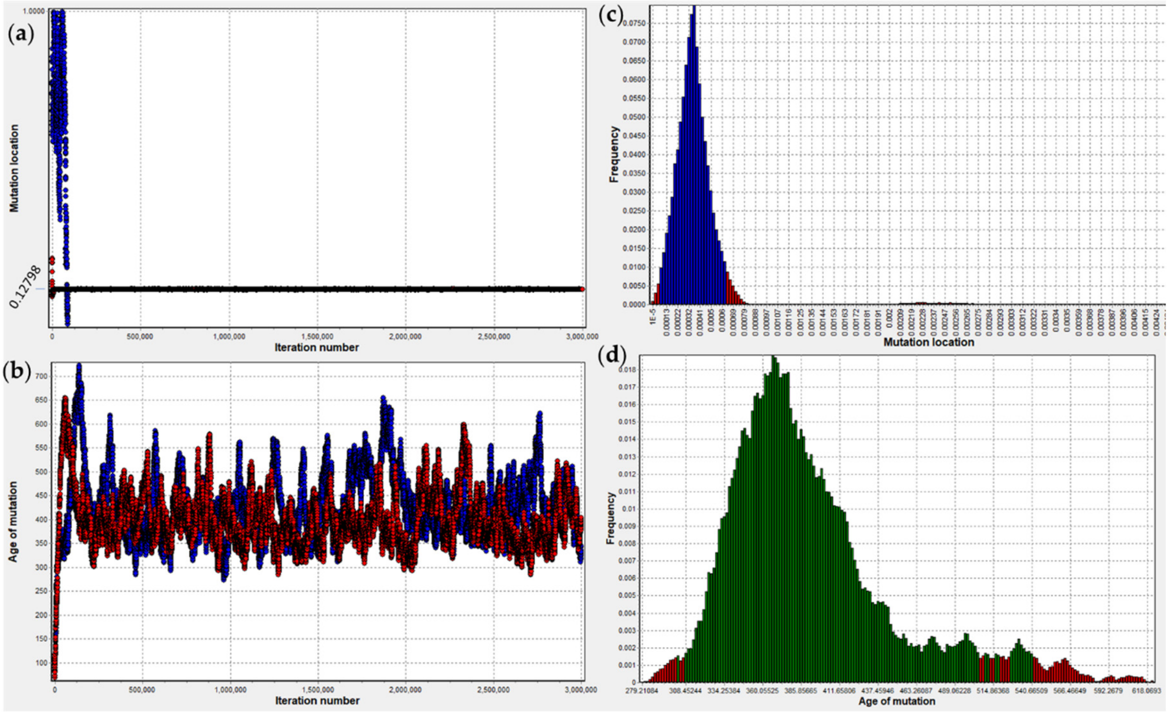

Figure S2: Estimation of p.(Arg143Trp) location and age for the unimputed data set ((a) and (b)) and the imputed data set ((c) and (d)). The two chains converged at the correct mutation location ((a) and (c)) while estimating the allele age ((b) and (d). In the unimputed set (b), Chain 2 stably estimated the allele age and this was apparent by its more precise estimation of the allele location from the very beginning of the burnin iterations (a).

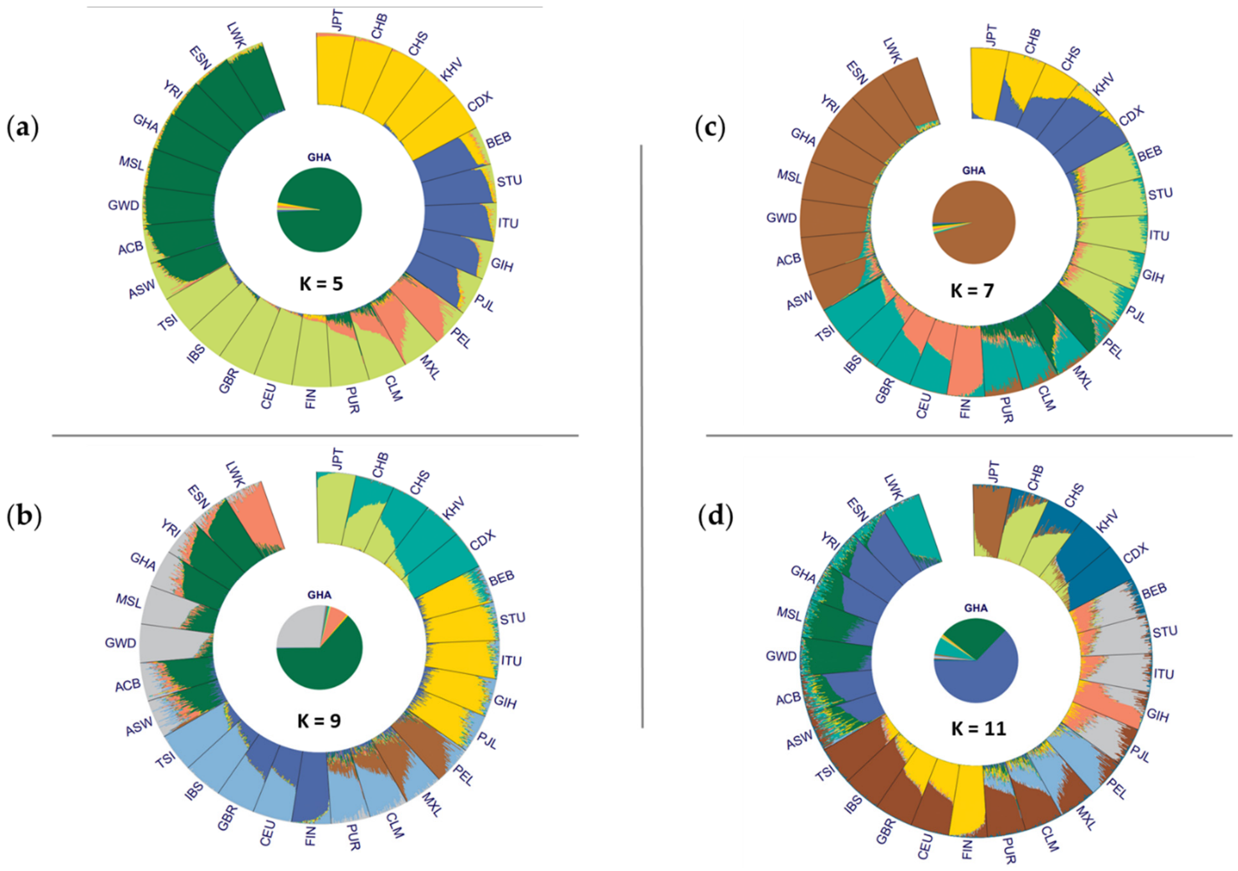

Figure S3: Ancestry analysis for Ghana and 1000 Genomes populations. At k = 5, all continental populations are resolved. European, American, as well as East Asian populations are further resolved at k = 7. From K = 8 to k = 11, all the continental ancestries are further resolved into sub-populations reflecting geographic separation. For instance, the LWK population in East Africa is clearly distinguished from all other African populations which are all from West Africa.
